# Research Progress of Photothermal Nanomaterials in Multimodal Tumor Therapy

**DOI:** 10.3389/fonc.2022.939365

**Published:** 2022-07-06

**Authors:** Xiaolu Shi, Ye Tian, Yang Liu, Zhengrong Xiong, Shaobo Zhai, Shunli Chu, Fengxiang Gao

**Affiliations:** ^1^ Department of Implantology, Hospital of Stomatology, Jilin University, Changchun, China; ^2^ University of Science and Technology of China, Hefei, China; ^3^ Changchun Institute of Applied Chemistry, Chinese Academy of Sciences, Changchun, China

**Keywords:** photothermal therapy, nanomaterial, tumor, multimodal therapy, photothermal agents, synergistic effect

## Abstract

The aggressive growth of cancer cells brings extreme challenges to cancer therapy while triggering the exploration of the application of multimodal therapy methods. Multimodal tumor therapy based on photothermal nanomaterials is a new technology to realize tumor cell thermal ablation through near-infrared light irradiation with a specific wavelength, which has the advantages of high efficiency, less adverse reactions, and effective inhibition of tumor metastasis compared with traditional treatment methods such as surgical resection, chemotherapy, and radiotherapy. Photothermal nanomaterials have gained increasing interest due to their potential applications, remarkable properties, and advantages for tumor therapy. In this review, recent advances and the common applications of photothermal nanomaterials in multimodal tumor therapy are summarized, with a focus on the different types of photothermal nanomaterials and their application in multimodal tumor therapy. Moreover, the challenges and future applications have also been speculated.

## 1 Introduction

Cancer poses a serious threat to human health worldwide, despite the developments in modern medical technology. Cancer is difficult or impossible to cure because it involves various genetic changes and cell abnormalities. Moreover, its complexity and heterogeneity promote the aggressive growth of cancer cells, resulting in significant incidence and mortality rates (1–3). The three traditional methods of tumor treatment include surgery, radiotherapy, and chemotherapy. However, due to severe surgical trauma, nonspecific and excessive radiation, and the irreplaceable defects of these therapies in targeting, bio-compatibility, multidrug resistance, and drug accumulation, patients may suffer from serious physiological side effects, resulting in poor quality of life and difficulty in achieving the target treatment effect (4–6).

These treatment deficiencies have inspired the development of new, precise, and more effective treatment strategies for tumors. For example, several emerging treatment methods, such as photodynamic therapy (PDT) (7, 8), photothermal therapy (PTT) (9, 10), and photoacoustic therapy (11, 12) have improved or can potentially improve therapeutic outcomes. Among them, PTT is a new type of minimally invasive tumor light therapy that has developed rapidly in recent years. It mainly uses photothermal conversion nanomaterials with strong absorption in the near-infrared light region (wavelength range 700–1300 nm) (13–15) to convert the absorbed light energy into heat energy effectively under the irradiation of the near-infrared laser, resulting in an increase in the temperature of local tumor tissues up to 40–45°C (hyperthermia) or above 45°C (thermal ablation) (14, 16). This results in degeneration and necrosis of tumor cells to achieve the goal of tumor therapy. The destruction of tumor tissue by PTT mainly occurs through killing tumor cells and destroying tumor blood vessels. The ability of tumor cells to tolerate high temperatures is much lower than that of normal cells. Specifically, the thermal lethal temperature of most tumor cells is between 42 and 43°C, while normal cells can tolerate such temperatures for a prolonged period. Therefore, the local hyperthermia produced by PTT can selectively kill tumor cells and cause irreversible damage, while normal cells are not damaged. Regarding blood vessels, compared with the blood vessels of normal tissue, those of tumor tissue have abnormal morphological growth, imperfect tissue and function, and are prone to rupture when the temperature and pressure increase. This causes tumor tissue to be more prone to damage by hyperthermia. Thus, PTT can effectively destroy tumor blood vessels, killing tumor cells without damaging normal tissues or causing systemic toxic reactions. Because PTT has the advantages of rapid targeted killing, being minimally invasive, and minimal toxic side effects, it is also known as “green therapy,” which carries significant potential in the field of alternative surgical resection (14, 17, 18).

It has been reported that PTT requires a temperature above 50°C to achieve tumor thermal ablation. In addition, cancer cells treated at low temperatures (around 43°C) may survive through self-repair of their heat shock proteins (HSPs), which could lead to treatment resistance and reduce treatment efficiency (19, 20). In recent years, the great progress in nanomaterials, medicine, and biology has promoted the application of nanomaterials in tumor therapy (21, 22). Moreover, great progress has been made in the construction of multifunctional photothermal nanomaterials, which can integrate a variety of treatment modes into a single nano platform. Compared with monotherapy, the combination of multiple therapies usually shows superiority in therapeutic effect. This advanced synergistic therapy can not only maintain the advantages of non-invasive, low toxicity, and convenient administration of PTT, but also relieve the problems of non-selectivity and multidrug resistance of traditional chemotherapy, and has achieved good therapeutic results (20).

This review will focus on the research progress of photothermal nanomaterials in multimodal tumor therapy and consists of a brief introduction to the classification of photothermal nanomaterials and their relative merits. Subsequently, multimodal treatments of tumors based on photothermal nanomaterials are clarified in detail. Finally, an outlook is provided to address recent challenges and suggest better treatment applications and research focuses to pursue new opportunities ahead.

## 2 Classification of Photothermal Nanomaterials

The goal of PTT is to make use of the hyperthermic effect of photothermal agents (PTAs), which can absorb light energy and convert it into heat energy, raising the temperature of the lesion site and ultimately causing the death of tumor cells (23, 24). To reduce the influence of localized high temperatures on normal tissue, near-infrared (NIR) light is usually selected for PTT because it has less tissue absorption and scattering and is able to penetrate deep tissue (15, 25). In addition, the ideal photothermal material should have higher photothermal conversion efficiency (PCE) and be accumulated effectively in tumor tissue (26). With the progress of PTT research and the rapid development of nanomaterials, photothermal nanomaterials have been more widely used than other photothermal materials because of their higher PCE and ability to be used in a multimodal tumor therapy platform (27–29). This review summarizes the common photothermal nanomaterials, which are divided into inorganic, organic, and composite photothermal nanomaterials (**Table 1**).

**Table 1:** Summary of photothermal nanomaterials in this review.

PTAs	Nanomaterials	Wavelength	PCE	Applications	Reference
Inorganic photothermal nanomaterials	aAuYSs	808nm	-	PTT+CT	(30)
	BSA-Silver NPs	690nm	–	PTT	(31)
	Pd nanosheets	808nm	-	PTT	(32)
	DPCN	808nm	–	PTT+CT	(33)
	USPIO-PEG-sLe^x^	808nm	-	PTT	(34)
	FA-BSA-PEG/MoOx@DTX	808nm	43.41%	PTT+CT	(35)
	WO_3_ nanosheets	808nm	41.6%	PTT	(36)
	Z@CD/P	808nm	–	PTT+CT	(37)
	mBMNI NPs	808nm	45.9%	PTT+PDT+CDT	(38)
	NB/CuS@PCM NPs	1060nm	–	PTT+CDT	(39)
	MoS_2_@DOX/MnO_2_-PEG	808nm	33.7%	PTT+CT	(40)
	TiS_2_ nanosheets	808nm 1064nm	46.82% (808nm) 45.51% (1064nm)	PTT+IT	(41)
	HMC-SS-PDA@CDs	808nm	35.9%	PTT+CT	(42)
	CdTeSe/ZnS@QDs	457nm	11%	PTT+PDT	(43)
	CNTs-PS/siRNA	808nm	59.3% (SCNT-PS) 57.8% (MCNT-PS)	PTT+GT	(44)
	HPP	1064nm	45.1%	PTT	(45)
	mGOG	808nm	-	PTT+CT	(46)
	DOX-Fe_3_O_4_@CGA	808nm	–	PTT+CT	(47)
	Nb_2_C@PDA-R837@RBC NPs	1064nm	27.6%	PTT+IT	(48)
	BP NS-PAMAM@DOX-HA	808nm	–	PTT+CT	(49)
	Co-P@mSio_2_@DOX-MnO_2_	808nm	-	PTT+CT	(50)
	UCNPs@mSiO_2_FePc-MC540	808nm		PTT+PDT	(51)
	PCM+PTX@mPBs/PEG	808nm	16,9%	PTT+CT	(52)
Organic photothermal nanomaterials	Cy5.5&ICG@ZIF-8-Dex	780nm	27.9%	PTT	(53)
	PPor NPs	808nm	70%	PTT+IT	(54)
	T-MPs	808nm	16.8%	PTT+Operation	(55)
	BBDP	690nm	54.2%	PTT+PDT	(56)
	DTPADPP/TPADDPP	635nm	48.1% (DTPADPP) 41.7% (TPADDPP)	PTT	(57)
	CMC/CS@PPy+5Fu NPs	808nm	21.6%	PTT+CT	(58)
	PANITG	808nm	55%	PTT+Starvation therapy	(59)
	NIRb14 NPs	808nm	31.2%	PTT	(60)
	MNP@PEDOT : PSS NPs	808nm	–	PTT	(61)
	Cu-PDA-FA NPs	808nm	46.84%	PTT+CDT+IT	(62)
	OMCNs	808nm	37.3%	PTT	(63)
	MNPs	808nm	87.65%	PTT+PDT	(64)
Organic–inorganic hybrid photothermal nanomaterials	Fe_2_O_3_@PEDOT-siRNA NPs	808nm	54.3%	PTT+GT	(65)
	RCDS@MIL-100	660nm	31.2%	PTT+CDT	(66)

PCE, photothermal conversion efficiency; PTT, photothermal therapy; CT, chemotherapy; CDT, chemodynamic therapy; IT, immunotherapy; GT, gene therapy.

### 2.1 Inorganic Photothermal Nanomaterials

#### 2.1.1 Precious Metal Nanomaterials

Precious metal nanomaterials, including gold, silver, palladium, and platinum, are considered to be simple and effective PTAs (23, 67, 68) due to their strong surface plasmon resonance (SPR), synthetic tunability, biological imaging potential, and excellent photothermal properties, such as high PCE in the high-absorption cross-section and NIR region.

Studies have shown that gold is one of the most popular nanomaterials for mediating PTT (69, 70), as it has good biocompatibility and low cytotoxicity (71). The photothermal conversion phenomenon in gold nanoparticles (GNPs) is based on the collective oscillation of free electrons on the surface of GNPs under electromagnetic radiation. The local area around GNPs is heated by electronic excitation and relaxation, which leads to the destruction of tumor tissue (72). At present, several gold nanomaterials with unique size and morphology have been developed, including nanorods, nanospheres, nanostars, nanocages, and nanoshells, among others. Choe et al. (30) loaded high concentration gold nanoparticles into mesoporous silica nanocapsules to form yolk-shell-structured gold nanospheres (aAuYSs) to study their photothermal effect on drug-resistant ovarian cancer cells. Under 808-nm laser irradiation, the cultured cancer cells were eliminated when the concentration of aAuYSs was 300 μg/mL. Moreover, *in vivo* experiments showed that after the combined treatment of aAuYSs and doxorubicin (Dox), the tumor volume and size were significantly reduced, and the number of Ki-67-positive proliferating cancer cells sharply decreased, indicating that aAuYSs can be used as a multifunctional photothermal nanoplatform for PTT and combined therapy.

Silver nanoparticles (SNPs), another type of precious metal nanomaterial, have been widely used due to their unique properties, such as controllable size and shape, easy modification, and excellent optoelectronic properties. Similar to GNPs, the SPR of SNPs can be adjusted to the infrared region by changing their size and shape (73). Additionally, Kim et al. (31) prepared SNPs coated with bovine serum albumin (BSA) (BSA-SNPs), which could internalize and kill melanoma cells by inducing ROS through cell analysis. These nanoparticles were also found to play a potential role in inhibiting angiogenesis. In addition, BSA-SNPs showed a significant increase in the temperature of a suspension under the irradiation of a laser at 690 nm and had a strong photothermal conversion capability, which could be used for photothermal cancer therapy.

Palladium-based nanomaterials, such as palladium nanosheets (74), porous/hollow palladium nanoparticles (75), and palladium@M (M=Ag, Au, Pt, SiO_2_, ZIF-8) (76–80) nanocomposites, also show strong absorption in the NIR region, as well as ideal PCE, excellent photothermal stability, and good biocompatibility (81). Therefore, palladium-based photothermal nanomaterials have become an option for cancer imaging contrast agents and therapeutic agents. Chen et al. (32) designed palladium nanosheets with a thickness of 1.8 nm and a diameter of 5–80 nm to evaluate the effect of size on the biological behavior of these nanosheets through cell and animal model experiments. The experimental results showed that compared with the large palladium nanowires, the smaller nanowires demonstrated a better photothermal effect under ultra-low laser irradiation. In addition, *in vivo* experiments revealed that 5-nm palladium nanosheets could escape the reticuloendothelial system with a longer blood half-life and be excreted from the kidneys, while the large nanosheets accumulated in the liver and spleen.

As a photothermal nanomaterial, platinum nanoparticles (PtNPs) slowly and continuously increase the temperature with light irradiation, not exceeding 46°C, which can effectively avoid normal cell damage (82, 83). Apart from good optical and photothermal stability, PtNPs can also be involved in the design of multimodal tumor treatment platforms, which can be used in combination with chemotherapy or radiotherapy (84, 85). Zhou et al. synthesized dendritic platinum-copper alloy nanoparticles (DPCNs) as a multimodal, therapeutic, tumor imaging platform (33). The PTT *in vitro* assay revealed that DPCNs ingested by PC-9 cells could effectively kill cancer cells under NIR irradiation. In addition, compared with the control group treated with DPCNs/NIR or Dox alone, the killing rate of cancer cells treated with DPCNs/Dox and irradiated with NIR laser was higher, indicating that DPCNs have potential for photothermal and chemotherapy.

#### 2.1.2 Transition Metal Dichalcogenide Nanomaterials

Transition metal dichalcogenides (TMDCs) are usually composed of one layer of transition metal atoms and two layers of chalcogenide atoms, and their generalized formula is MX_2_. M refers to the transition metals of groups 4–10, such as copper, molybdenum, tungsten, titanium, etc., while X refers to a chalcogen (86). It has been found that monolayer TMDCs exhibit strong NIR absorption, good PCE, and excellent photothermal stability (87, 88), giving TMDCs the potential to be used as PTAs (89).

In recent years, copper nanomaterials have been widely used in cancer therapy (90). Among those used in PTT for cancer, such as copper selenide, copper telluride, and copper oxide, copper sulfide is the most explored (91). It has been found that as a P-type semiconductor, copper chalcogenide nanomaterials have composition-dependent localized SPR and ideal PCE in the NIR region (92, 93). Moreover, Huang et al. (39) combined monoterpenoid sensitizer, borneol, and NIR-II PTA copper sulfide to make thermo-responsive vehicle NB/CuS@PCMNPs. Under the irradiation of a 1060-nm laser, the high temperature produced by copper sulfide nanoparticles can be used in PTT. The results of animal experiments showed that NB/CuS@PCMNPs could aggregate in the tumor site and significantly inhibit tumor growth.

Titanium disulfide is another common material for TMDCs with excellent stability, electrical conductivity, and strong absorption in the NIR window (41). In addition, due to the local SPR effect, the absorption peak of these nanosheets can be shifted from red to the range of 1000–1350 nm by adjusting the thickness and width of the nanowires (94). Fu et al. (41) made a multifunctional NIR-II nano-preparation based on titanium disulfide, which can be used in magnetic targeted NIR-II photoacoustic/magnetic resonance imaging-guided synergistic photothermal-immune combination therapy. The results of *in vivo* experiments showed that the primary tumors in the group that underwent PTT combined with immunotherapy disappeared without recurrence after 16 days of treatment. This significantly inhibited the tumor growth rate, indicating that titanium disulfide has great potential in the field of PTT combined with immunotherapy and imaging.

In addition, the crystal structure of molybdenum disulfide is a honeycomb, similar to graphene, which can be obtained through stripping or synthesis and has a variety of forms, such as nanosheets and quantum dots, among others (95–98). It has been found that molybdenum disulfide nanoparticles have become commonly used PTAs in cancer treatment due to their good biocompatibility, strong SPR, excellent PCE, and low production cost (99). Liu et al. (40) synthesized a mesoporous core-shell structure with molybdenum disulfide as the core and manganese dioxide as the shell. This structure was used to wrap the chemotherapeutic drug, Dox, and then modified with mPEG-NH_2_ to prepare MoS_2_@Dox/MnO_2_-PEG (MDMP) composite antitumor nanocomposites. The *in vivo* and *in vitro* experiments showed that MDMP had excellent antitumor activity (tumor survival rate: 11.8%) and good PCE (33.7%).

#### 2.1.3 Metal Oxide Nanomaterials

In addition to TMDC nanomaterials, nanomaterials containing transition metal oxides have also received extensive attention in the field of PTT (100) due to their excellent PCE good biocompatibility, excellent chemical stability, adjustable band gap, and low cost. Iron oxide, molybdenum oxide, tungsten oxide, zinc oxide, and manganese oxide are used as common metal oxide nanomaterials.

**Figure 1:**
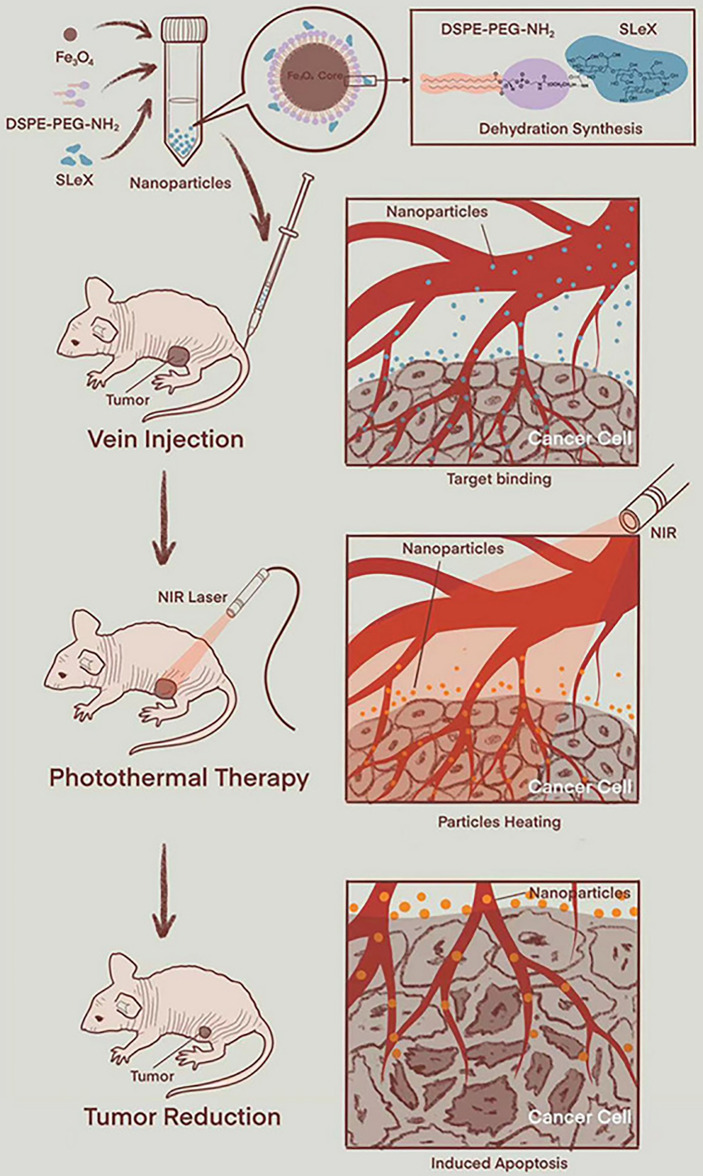
Schematic diagram of PTT (using animal experimental research as a model) (34). Reproduced with permission from Liu et al., 2021.

Magnetic nanoparticles, mainly including magnetite (Fe_3_O_4_), maghemite (γ-Fe_2_O_3_), or a combination of the two (101) show great potential in cancer therapy in the form of magnetic resonance imaging-guided chemotherapy (102, 103), PDT (104, 105), and PTT (106, 107) due to their unique superparamagnetic iron oxide nanoparticles. In addition, iron oxide nanoparticles show excellent PCE in a biological environment and have good chemical stability and low cytotoxicity (108). Moreover, the US Food and Drug Administration has approved its application in the human body (109). Liu et al. (34) synthesized USPIO-PEG-sLe^x^, which consists of nanocomposites of ultrasmall superparamagnetic iron oxide nanoparticles coated with polyethylene glycol (PEG) coupled with Sialyl Lewis X. The USPIO-PEG-sLex nanoparticles have good photothermal conversion properties, and the temperature and concentration of the solution are positively correlated with the power density of NIR on 808-nm wavelengths. The results of PTT *in vitro* showed that as the nanoparticle concentration increased, the survival rate of 5-8F cells significantly decreased, which could effectively inhibit the development of tumors (**Figure 1**).

**Figure 2:**
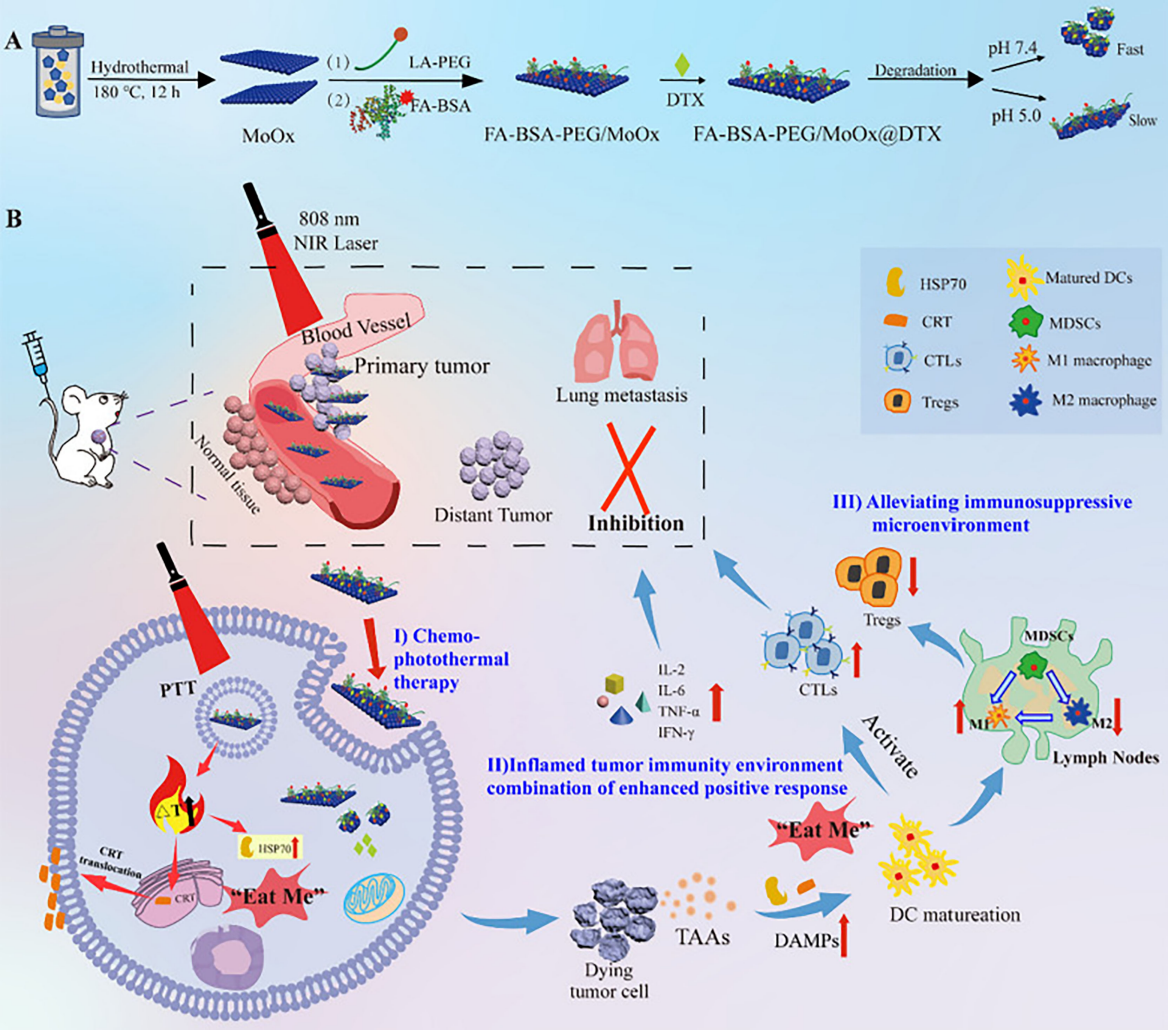
The schematic illustration of the multi-strategy for cancer treatment. **(A)** The preparation route of the FA-BSA-PEG/MoOx@DTX nanosheet and the *in vitro* antitumor and degradation experimental design; **(B)** the elucidation of the mechanism of FA-BSA-PEG/MoOx@DTX + NIR combination therapy for meliorating tumor immunosuppression, inhibiting distant tumor and lung metastasis (35). Reproduced with permission from (35).

Molybdenum-based materials can be divided into two categories: transition metal oxides composed of molybdenum dioxide and molybdenum trioxide, and TMDCs composed of molybdenum disulfide. Transition metal molybdenum oxide has a similar, adjustable, local SPR effect to precious metal nanomaterials (110). Molybdenum trioxide nanoparticles have been reported to have excellent light absorption ability in the NIR region and can produce singlet oxygen under the irradiation of NIR light. Thus, molybdenum oxide nanomaterials can be used in PDT or PTT for tumors (111, 112). Qiu et al. (35) combined folic acid and α-lipoic acid-conjugated mPEG-NH_2_ (LA-PEG) and modified BSA with molybdenum oxide nanosheets to prepare multi-functional degradable FA-BSA-PEG/MoOx nanosheets (**Figure 2**). The results of *in vivo* and *in vitro* anti-tumor experiments showed that FA-BSA-PEG/MoOx nanosheets significantly increased the temperature of the tumor site, inducing immunogenic cell death, which triggered an immune response *in vivo* through the combination of PTT and chemotherapy, inhibiting primary tumor growth (inhibition rate: 51.7%) and lung metastasis (inhibition rate: 93.6%). This novel nanosheet is a promising avenue for combination therapy for breast tumors.

Tungsten nanomaterials, similar to precious metal nanomaterials, exhibit a local SPR effect because of their outer-d valence electrons, which can be used to produce a photothermal effect (113). The size and shape of the nanoparticles also play a role in the SPR effect of tungsten nanomaterials (114, 115). Among tungsten nanomaterials with different stoichiometry, tungsten oxide nanomaterials are among the most widely used materials in the biomedical field (116, 117). In particular, W_20_O_58_, W_18_O_49_, and W_24_O_68_ are more common PTAs because of their excellent optical absorption capacity in the NIR region (118). Liang et al. (36) introduced oxygen vacancy (OV) tuning into oxygen-deficient tungsten trioxide nanosheets to optimize the chemical and electrical properties. The experimental results show that under the irradiation of single-wavelength NIR (808 nm), tungsten trioxide-OVs exhibited good PCE (41.6%) and an effective tumor inhibition rate (96.8%).

Zinc oxide is a multi-functional material with unique physical and chemical properties, such as high chemical stability, high electrochemical coupling coefficient, wide radiation absorption range, and high light stability (119, 120). Zinc oxide nanomaterials can appear in one-dimensional (121), two-dimensional (122), and three-dimensional (123) structures, providing one of the greatest assortments of particle structures among all known materials (124). Thus, zinc oxide is a potential alternative for PTT (72). Deng et al. (37) prepared multifunctional nanoparticles (Z@CD/P) using ZnO@CuS as the carrier, as well as β-cyclodextrin (β-CD) modified by 2,3-dimethyl maleic anhydride (DMA) (β-CD-DMA), and mPEG-NH_2_ modified by DMA (PEG-DMA) to increase stability. They were loaded with Dox and pirfenidone (PFD). Zinc oxide and copper sulfide were found to promote tumor cell death by regulating the pathway of ROS production as well as that of GSH-GPX_4_, and their photothermal conversion ability further promotes the anti-tumor effect.

**Figure 3:**
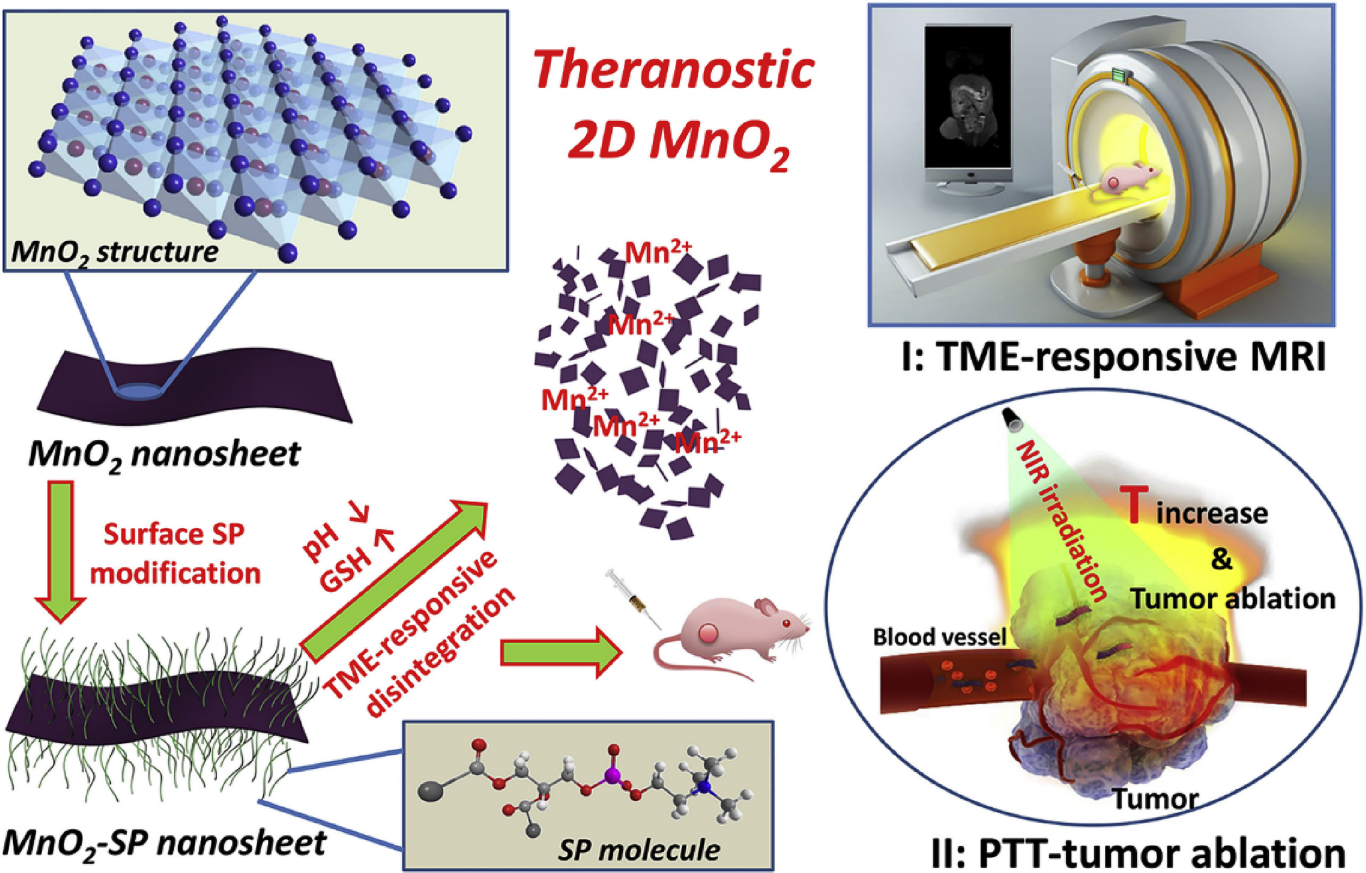
Schematic illustration of synthetic procedure for MnO2-SPs nanosheets and their specific functions for tumor theranostics with TME sensitivity, including the acidic/reducing condition-triggered T1-weighted MR imaging and efficient PTT against tumor (126). Reproduced with permission from (126).

Manganese oxide nanomaterials have great potential as PTAs and signal contrast agents for traditional PTT because of their excellent T1-weighted contrast signals, low cytotoxicity, and high PCE (72, 125). Liu et al. (126) proposed for the first time that ultra-thin manganese dioxide nanosheets have pH and redox responses as well as T1-weighted magnetic resonance imaging capabilities. Moreover, photothermal *in vivo* and *in vitro* experiments showed that these nanosheets also had good photothermal conversion ability (η: 21.4%) and a high inhibition rate on tumor growth (**Figure 3**). Xu et al. (38) designed bismuth/manganese oxide nanoparticles (mBMNI NPs) for targeting triple-negative breast cancer, which were encapsulated in the tumor cell membrane and loaded with indocyanine green. The result of the photothermal experiment showed that mBMNI NPs absorbed NIR laser efficiently and generated a large amount of heat for PTT. Apart from high-efficiency PTT, mBMNI NPs also performed chemodynamic therapy (CDT) and PDT synergistically through the generated singlet oxygen and ICG, offering great potential for targeted triple-negative breast cancer therapy.

#### 2.1.4 Carbon-Based Nanomaterials

In recent years, carbon-based nanomaterials have been widely studied as inorganic materials for PTT for tumors (100). Many carbon-based nanostructures have been developed for biomedical applications, such as carbon dots, quantum dots, graphene, and carbon nanotubes, among others. The graphitic structure of carbon-based materials endows them with strong absorption in the NIR region and good PCE (127, 128). In addition, the ultra-high surface area of carbon-based materials enables them to build multifunctional nanoplatforms, which have optimistic application prospects in tumor therapy (129).

##### 2.1.4.1 Carbon Dots

As a new type of 0 dimensionality material, carbon dots (CDs) not only inherit the advantages of small molecules (such as fluorophores) and traditional semiconductors (such as inorganic quantum dots), but they also have additional properties (130). For example, CDs have excellent photostability, good biocompatibility, permeability, low toxicity, low cost, and are easy to prepare (131). However, most CDs usually absorb light in the short wavelength region due to the π-π* transition of the C=C bond; therefore, other nanomaterials, such as metal nanoparticles (132) or semiconducting polymers (133) are needed as NIR-assisted PTAs (131). Lu et al. (42) assembled polydopamine (PDA) and carbon points on hollow mesoporous carbon (HMC) to construct a photothermal enhanced multi-functional system (HMC-SS-PDA@CDs). The results of *in vivo* experiments showed that under low-power, 808-nm laser radiation of 0.75 W/cm^2^, the antitumor drug-loaded HMC-SS-PDA@CDs inhibited tumor growth by 92.6% and significantly reduced the toxicity of Dox to cells, indicating that Dox/HMC-SS-PDA@CD nanoparticles have good photothermal chemotherapeutic synergism and ideal biocompatibility.

##### 2.1.4.2 Quantum Dots

Compared with traditional fluorescent dyes and proteins, quantum dots (QDs) have significant advantages, such as broad luminescence excitation spectra and narrow symmetrical emission spectra with large Stokes shifts (134, 135). Different types of quantum dots have varying chemical compositions and properties, which can affect their potential applications. The new generation of quantum dots, including non-cadmium and NIR-II window quantum dots, have excellent optical properties and biocompatibility required for *in vivo* applications and good prospects in the field of tumor therapy and imaging (136–138). Wang et al. (43) have developed cadmium tellurium selenium/zinc sulfide core-shell quantum dots with excellent biocompatibility for PTT and fluorescence imaging of tumors. After being irradiated with blue light (80 mW/cm^2^ blue laser) for 20 min, the quantum dots were heated rapidly. Due to their photothermal and photodynamic effects, the quantum dots induce complete apoptosis of the Huh7 hepatoma cell line, providing a new avenue for tumor therapy.

##### 2.1.4.3 Carbon Nanotubes

Carbon nanotubes (CNTs), originally proposed by Iijima (139), are currently the most widely used carbon-based nanomaterials in the biomedical field (140, 141). CNTs are divided into two types according to the number of layers in their structure: single-walled CNTs (SCNTs), which consist of a single graphene sheet, and multi-walled CNTs (MCNTs), which consist of several sheets forming concentric cylinders (142). CNTs have been reported to have broad NIR absorption and are affected by the size and shape of the nanomaterials (143). CNTs exposed to NIR laser absorbs light energy and converts it into thermal energy, which can be used to ablate cancer cells (144). Zhao et al. (44) coated SCNTs and MCNTs with peptide lipid and sucrose laurate, respectively and loaded anti-survivin siRNA to synthesize a nano-delivery system (denoted SCNT-PS and MCNT-PS, respectively) with good temperature sensitivity and photothermal properties for tumor immunity and combination PTT. The results showed that CNT/siRNA inhibited tumor growth by silencing the expression of survivin and exhibiting a photothermal effect under NIR laser. SCNT-PS/siRNA showed high antitumor activity and had a complete inhibitory effect on some tumors. Neither SCNT-PS nor MCNT-PS nanoparticles had obvious cytotoxicity at a concentration of up to 60 μg/mL.

##### 2.1.4.4 Mesoporous Carbon Nanoparticles

Mesoporous carbon nanoparticles (MCNs) or hollow carbon nanospheres (HCNs) are mesoporous nanomaterials with high pore volume and specific surface area, which have attracted attention in recent years (145, 146). It has been found that MCNs have a unique structure that can load a large number of hydrophobic drugs as well as excellent biocompatibility, which makes them an effective drug carrier (147, 148). In addition, MCNs have high efficiency in converting NIR laser energy into thermal energy and can be used in tumor PTT (72, 149). Xu et al. (45) designed polyethylene glycol-graft-polyethylenimine (HPP)-modified HCNs as NIR-II responsive PTAs. The experimental results showed that HPP-HCNs have a PCE of 45.1% under 1064nm laser irradiation. The *in vivo* and *in vitro* experiments showed that HPP had limited cytotoxicity to mice and good photothermal activity towards killing cancer cells in the xenograft 4T1 tumor-bearing mice model, which significantly inhibited tumor growth.

##### 2.1.4.5 Graphene-Based Nanomaterials

Graphene, as a common carbon-based nanomaterial, has a wide range of applications as a biosensor, drug carrier, and tumor PTA because of its strong NIR absorption (150, 151). However, graphene has poor dispersibility in physiological fluid and is considered to have certain biological toxicity (152). To overcome these limitations, graphene requires surface modification with specific materials (153). Therefore, graphene-based nanomaterials (GBNs) have received increasing attention (154–156). For example, GBNs have been found to have a large surface area and can be used as drug carriers (157). GBNs are also widely used as PTAs in tumor therapy because of their good photothermal conversion ability in the NIR region (158, 159). Generally, GBNs can be divided into several types, including graphene with varied layers, graphene oxide (GO), and reduced graphene oxide (rGO) (160).

GO and rGO have great potential in the field of biomedicine, especially in drug delivery, biosensors, and targeted tumor therapy because of their tunable physicochemical properties, excellent biocompatibility, and outstanding photothermal properties (160–163). Dash et al. (46) modified rGO with citrate-coated magnetic nanoparticles, coupled with gastrin-releasing peptide receptor-binding peptide, and loaded Dox through the π-π bond to synthesize an rGO-based magnetic nanocomposite (mGOG). The results of the *in vitro* experiments showed that after being combined with 808-nm laser irradiation, the 50% inhibiting concentration and apoptosis rate of tumor cells were 0.19 μg/mL and 76.8%, respectively. At the same time, the increased expression of heat shock protein HSP70 confirmed the magnitude of the photothermal effect of mGOG. In addition, the mouse model experiment showed that, after 5 min of NIR laser irradiation, the tumor volumes in the mice in the experimental group were significantly reduced, the survival time was significantly prolonged, and the antitumor effect was significant.

With the continuous study of GBNs, graphene quantum dots (GQDs) first discovered by Ponomarenko and Geim (164), have undergone vigorous development in the biomedical field. GQDs exhibit inherent fluorescence properties, low cytotoxicity, stable photoluminescence, good biocompatibility, and superior resistance to photobleaching (165). After NIR light irradiation, GQDs also show excellent photothermal conversion ability (166, 167). These unique physicochemical properties endow GQDs with excellent potential in tumor therapy. Chen et al. (47) combined aptamer-modified GQDs with magnetic chitosan to form novel photothermal-chemotherapy drug delivery nanosystems (DOX-Fe_3_O_4_@CGA). The results of an *in vivo* antitumor experiment showed that under NIR laser irradiation, the temperature of the tumor site in mice increased rapidly to 43–45°C, and the tumor volume and weight significantly decreased over time. Thus, DOX-Fe_3_O_4_@CGA significantly inhibited tumor growth and prolonged survival time in mice, demonstrating excellent synergistic therapeutic ability.

#### 2.1.5 Other Inorganic Photothermal Nanomaterials

**Figure 4:**
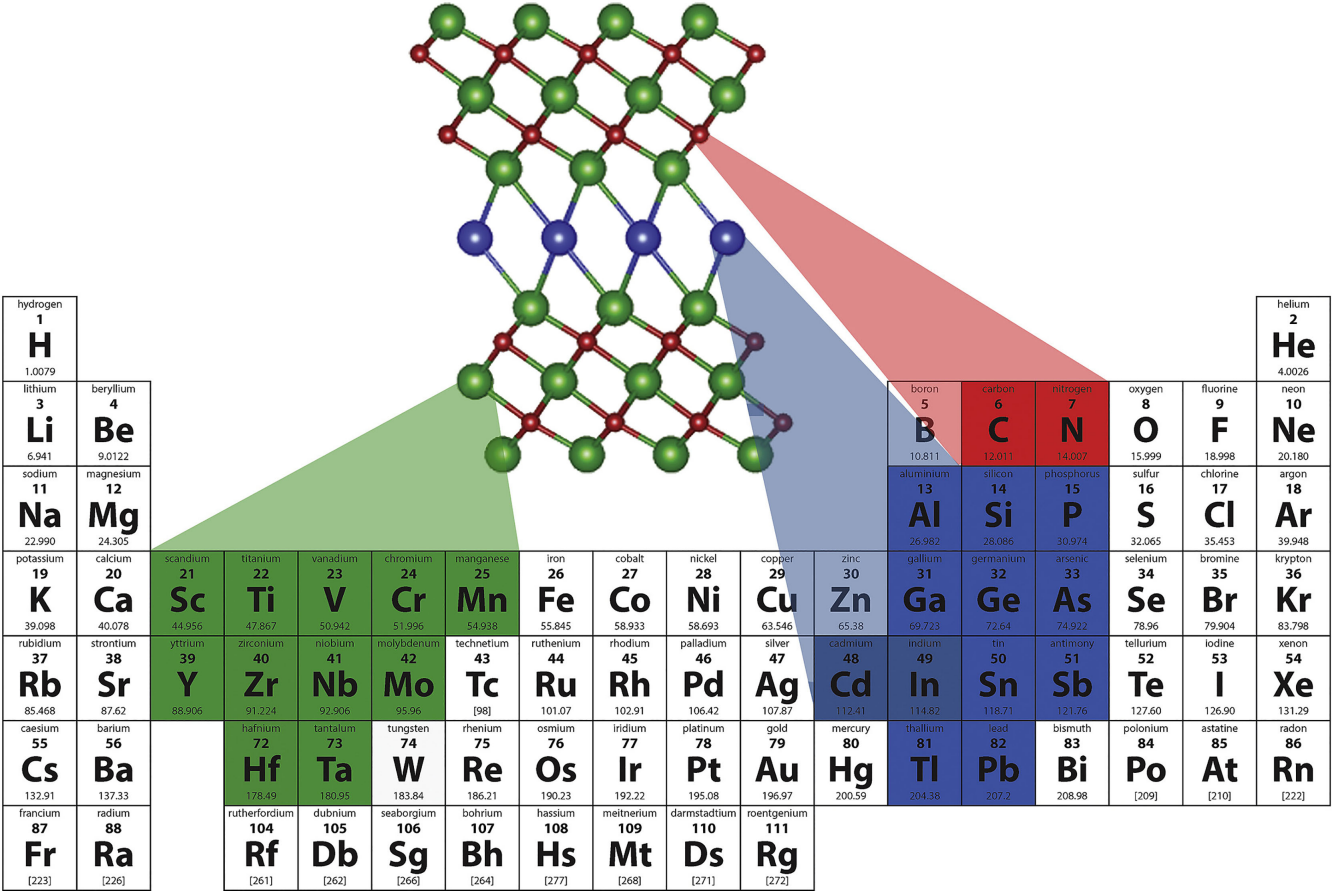
MAX phases Mn+1AXn forming elements (171). Reproduced with permission from (171).

MXenes refer to a series of carbides, nitrides, and carbonitrides containing transition metals (mainly from groups 3 and 4), with unique structure and excellent physicochemical properties (168–170) (**Figure 4**). The typical molecular formula is expressed as Mn+1X_n_T_x_ (e.g, Ti_3_C_2_T_x_). Notably, MXenes have good optical properties for bioimaging and biosensors, and their excellent PCE and biocompatibility make them ideal candidates as efficient PTAs (172, 173). Lu et al. (48) coated a layer of red blood cell membrane on polydopamine-modified niobium carbide nanosheets coated with immunoadjuvant R837 to synthesize a new type of multifunctional niobium carbide nanoparticle (Nb_2_C@PDA-R837@RBCNP) for NIR-II PTT combined with immunotherapy. Nb_2_C@PDA-R837@RBCNPs exhibited high PCE under 1064-nm laser irradiation. Additionally, the circulation time *in vivo* was significantly prolonged, and the primary tumors were completely cleared in mice. Finally, the secondary tumor growth inhibition rate was as high as 89.8% due to the enhanced immune response.

Compared with other two-dimensional nanomaterials, black phosphorus nanosheets (BPNSs) have a larger extinction coefficient and higher PCE and are often used as PTAs for PTT (174–176). In addition, BPNSs have been widely used in biomedicine because of their large specific surface area, good biocompatibility, and biodegradability (177). Peng et al. (49) prepared BPNS-based multifunctional nanocomposites (BPNS-PAMAM@DOX-HA) by modifying BPNSs with hyaluronic acid and poly-amidoamine dendrimer and loading them with Dox. The results of the *in vivo* and *in vitro* experiments showed that BPNS-PAMAM@DOX-HA exhibited excellent tumor cytotoxicity and cellular uptake efficiency under 808-nm laser irradiation, significantly inhibited the growth of tumors in mice, and showed a more significant antitumor effect than chemotherapy or PTT alone.

Metal phosphorus-based nanomaterials (metal-PNMs) mainly include metal phosphide nanomaterials (e.g., ferrous phosphide) (178), metal phosphate nanomaterials (e.g., calcium phosphate) (179), and metal-black phosphorous nanocomposites (180). Among PNMs, metal-PNMs have been widely studied for tumor diagnosis and treatment due to their unique advantages such as excellent light absorption, inherent magnetism, and biodegradability (181). Jin et al. (50) created a novel anticancer nanoplatform (Co-P@mSiO_2_@DOX-MnO_2_) for the synergistic treatment of tumor chemotherapy and PTT, which used cobalt phosphide nanocomposite as the core and mesoporous silica as the shell, loaded with Dox, and combined with manganese dioxide nanosheets. The results showed that under the irradiation of 808-nm NIR laser, Co-P@mSio_2_@DOX-MnO_2_ rapidly increased the temperature of the tumor, reflecting the excellent photothermal conversion ability. Compared with the control group, the tumor growth inhibition of the Co-P@mSio_2_@DOX-MnO_2_ group was greater and the antitumor effect was significantly improved.

In recent years, upconversion nanoparticles (UCNPs) have attracted attention for their ability to convert NIR light into visible light or ultraviolet light with a shorter wavelength (182). UCNPs have the advantages of minimizing light damage, deep tissue penetration, low light bleaching, and good chemical stability (183–185), which give them great potential for application in tumor therapy (186). Zhang et al. (51) designed a lanthanide-doped UCNP nanotherapy platform (UCNPs@mSiO_2_FePc-MC540) coated with mesoporous silica for synergistic PDT and PTT, which included NaYF_4_:Yb, Er@NaLuF_4_:Nd@NaLuF_4_ UCNPs, and dual photosensitizing agents (merocyanine 540 and iron phthalocyanine). The results of the antitumor experiment showed that the survival rate of A549 cells in the UCNPs@mSiO_2_FePc-MC540 group decreased significantly under 808-nm light, while the tumor volume decreased to approximately 10% of the original volume, showing a significant antitumor effect.

Prussian blue (PB) is an iron-centered compound (Fe_4_[Fe(CN)_6_]_3_-xH2O, where x is the number of water molecules), which has been widely studied as a coordination compound (187, 188). PBNPs are widely used in immunosensors, biological imaging, drug release, and tumor therapy due to their large inner pore volume, adjustable size, easy synthesis, surface modification, good thermal stability, and biocompatibility (189–193). Liu et al. (52) mixed paclitaxel (PTX) and phase change materials (PCM) and loaded them onto polyethylene glycol-modified mesoporous PBNPs (mPBs) to construct a biocompatible nano-drug delivery system (PCM+PTX@mPBs/PEG). The *in vitro* cell experiment showed that the cellular uptake rate of PCM+PTX@mPBs/PEG increased significantly after 808-nm NIR laser irradiation. The *in vivo* antitumor experiment showed that PCM+PTX@mPBs/PEG could accumulate in the tumor site of mice by passive transport and significantly inhibit tumor growth by delivering chemotherapeutic drugs and a photothermal effect.

### 2.2 Organic Photothermal Nanomaterials

Inorganic photothermal nanomaterials are easy to prepare and highly modifiable (141), and tend to have higher PCE and better photothermal stability than organic nanomaterials (26, 194). However, the potential cytotoxicity caused by the poor biodegradability of inorganic materials hinders their clinical application (195). In contrast, organic photothermal nanomaterials are more biodegradable and biocompatible (26, 196) and mainly include organic small-molecule nanomaterials and conjugated polymer nanomaterials (14, 197). These two types of PTAs have shown good therapeutic effects and are frequently used for tumor imaging and treatment (198, 199). The most common organic photothermal nanomaterials are introduced below.

#### 2.2.1 Organic Small-Molecule Nanomaterials

Common organic small-molecule photothermal materials include cyanine dyes, porphyrins, phthalocyanines, boron dipyrromethene, and diketopyrrolopyrrole (DPP). Although these small molecules have excellent photothermal conversion ability and biosafety, they also have limitations, such as poor water solubility and limited tumor accumulation. Through functional modification, nanocarriers can be designed to improve the solubilization and pharmacokinetics of small organic molecules and enhance the penetration and retention of therapeutic agents in tumor tissue, enhancing the therapeutic effect (26).

After modification to improve the photophysical properties, cyanine dyes are widely used in tumor PTT, imaging, and sensing because of their excellent biocompatibility and strong NIR absorption (200, 201). Cyanine molecules such as ICG, IR825, IR780, and cypate, are common PTAs that show potential for widespread application in fluorescence imaging and tumor therapy (26, 202). Guo etal. (53) synthesized zeolitic imidazolate framework-8 (ZIF-8) composite nanoparticles (Cy5.5&ICG@ZIF-8-Dex) using dimethyl sulfoxide/water solvent mixtures and loaded ICG and cyanine-5.5 (Cy5.5) for tumor imaging and PTT. The results of PTT showed that the A549 cells in the Cy5.5&ICG@ZIF-8-Dex group died in large numbers, and the tumor growth rate in mice was significantly inhibited, achieving an excellent therapeutic effect.

Porphyrin-based nanomaterials, with good photophysical properties and biocompatibility, have gained extensive attention in clinical tumor therapy and diagnostic imaging (203, 204). Studies have found that assembling porphyrin monomers with supramolecular nanostructures not only improves their physical and chemical properties and strengthens tumor accumulation, but also greatly enhances the range of application of porphyrin in the biomedical field (205, 206). Cao et al. (54) synthesized amphiphilic porphyrin (PPor) through conjugation with two PEG chains, and integrated perylene diimide into the porphyrin skeleton to form a D-A structure. The *in vivo* and *in vitro* antitumor experiments showed that under 808-nm laser irradiation, PPor nanoparticles completely disappeared from the primary tumor in mice and stimulated robust systemic antitumor immunity by releasing a large number of damage-associated molecular patterns and tumor-associated antigens, which significantly inhibited tumor metastasis.

Phthalocyanines (PCs) are regarded as second-generation photosensitizers in PDT because of their high molar absorption and excellent photostability (202, 207). With further research on PCs, it was found that PC nanomaterials also exhibit high PCE after irradiation with NIR light, giving them great potential in the application of PTT (208, 209). Feng et al. (55) designed T-MP nanoplatforms based on HER2 and targeted micellular PC. The results of *in vivo* and *in vitro* antitumor experiments showed that after 808-nm laser irradiation, the killing rate of HT-29 cells in the T-MP group was much higher than that in the control group. Additionally, primary tumor growth was significantly suppressed, and tumor lymph node metastasis was effectively overcome, greatly prolonging the survival time of mice.

Compared with other organic photothermal nanomaterials, boron dipyrromethene (BODIPY) has gained interest because of its strong absorption of long wavelengths, good photostability, excellent water solubility, and biocompatibility (210–214). Through chemical modification with a conjugated system, the nano-photosensitizer based on BODIPY has a higher absorption coefficient in the NIR region, which gives it the potential to be used as a PTA (215–217). Yu et al. (56) fabricated an NIR BODIPY dye with an upper phenyl-fused segment (BBDP). The photothermal experimental results demonstrated the PCE of BBDP nanoparticles to be as high as 54.2%, suggesting excellent photothermal capability. Moreover, *in vitro* antitumor experiments showed that under 690-nm laser irradiation, as the concentration of BBDP-NPs increased, the survival rate of tumor cells gradually decreased, indicating that BBDP-NPs have a good phototherapeutic effect.

DPP and its derivatives are widely used in fluorescence imaging and tumor therapy because of their easy modification, high molar extinction coefficient, and good photothermal stability (218, 219). In recent years, nanomaterials with a D-A-D structure based on DPP derivatives have received increasing attention (220, 221). Zheng et al. (57) synthesized three self-assembled nanoparticles with PEG as the side chain using three amphiphilic DPP derivatives (TPADPP, DTPADPP, and TPADDPP). The experimental results showed that these three nanoparticles can not only effectively gather in the tumor site, but also have good biological safety and low cytotoxicity in dark environments. Under the irradiation of a 635-nm laser, DTPADPP and TPADDPP nanoparticles showed an efficient photothermal effect, and tumor growth in mice was significantly inhibited, suggesting that they have a tumor ablation effect.

#### 2.2.2 Conjugated Polymer Nanomaterials

Conjugated polymers with a large π-conjugated backbone and high electron delocalized structure have been widely used in tumor therapy because of their high extinction coefficient and good biocompatibility (222, 223). Moreover, because of their π-electrons, which can easily cause delocalization and transition, conjugated polymers can effectively convert absorbed light energy into heat, making it a suitable PTA (224). At present, the conjugated polymer nanomaterials mainly include polypyrrole (PPy), polyaniline (PANI), and donor-acceptor (D-A)-conjugated polymers, as well as poly-(3,4-ethylenedioxythiophene): poly(4-styrene sulfonate) (PEDOT : PSS).

As a potential PTA, PPy has good biocompatibility, excellent photothermal properties, photostability, and accessible synthesis characteristics (225, 226). Wang et al. (58) combined CS and carboxymethyl cellulose (CMC) through electrostatic interactions and loaded PPy and 5-fluorouracil (5-Fu) to prepare a novel composite nanoparticle: CMC/CS@PPy+5FuNP. *In vitro* biological studies showed that CMC/CS@PPy+5FuNPs can be effectively internalized by HepG2 cancer cells. The combination of the photothermal effect of PPy and toxicity of 5-FU can significantly improve the therapeutic efficiency on tumors, indicating that CMC/CS@PPy+5FuNPs have great potential in synergistic chemotherapy and PTT.

PANI has been reported as a type of organic photothermal nanomaterial with good stability, biocompatibility, and strong NIR absorptivity. It has been widely used in photoacoustic imaging (PAI) and PTT for tumors (227–229). Wu et al. (59) synthesized a glucose oxidase (GOx)-conjugated PANI nanoplatform (PANITG) for PTT for tumors. Under the irradiation of NIR laser, PANITG activates PTT in slightly acidic tumor microenvironments. The released GOx reacts with excess glucose in the tumor tissue, resulting in cancer starvation. The *in vitro* and *in vivo* antitumor experiments showed that glutamate produced by GOx-mediated catalytic reactions enhances the photothermal effect. Meanwhile, PTT also plays a role in promoting the catalytic reaction, indicating that the two synergistically exhibit a significant antitumor effect.

D-A-conjugated polymers have been widely used in PTT for tumors because of their extended light absorption ability and good PCE (230, 231). Liu et al. (60) synthesized D-A-conjugated nanoparticles using thiophene and triphenylamine (TPA) as donors and benzo[1,2-c:4,5-c′]bis([1,2,5]thiadiazole) (BBTD) as the acceptor. The molecular rotors and bulky alkyl chains were then introduced into the center Dmura core to reduce intermolecular interaction. NIRb14 nanoparticles (NIRb14NPs) with long alkyl chains exhibit better photothermal properties. Additionally, the *in vivo* and *in vitro* antitumor experiments showed that NIRb14NPs had a longer circulation time *in vivo*, demonstrating significant tumor growth inhibition and biosafety.

Poly(3,4-ethylenedioxythiophene): poly(styrene-sulfonate) (PEDOT : PSS) is a complex of conjugated polymer PEDOT and negatively charged polymer PSS (232–234). PEDOT : PSS is an aqueous-based conductive polymer nanoparticle with strong NIR absorption that has become a popular NIR PTA for its water-dispersibility, high PCE, excellent light stability, and good biocompatibility (235–237). Ko et al. (61) synthesized a kind of magneto-conjugated polymer core-shell nanoparticle (MNP@PEDOT : PSSNP) based on PEDOT : PSS by *in situ* surface polymerization. PTT experiments showed that after laser irradiation, compared with the control group, the tumor volume of tumor-bearing mice did not significantly change, while the tumors in the MNP@PEDOT : PSSNP group were completely cleared. Additionally, these nanoparticles did not affect other organs, showing an effective and safe anti-tumor effect.

**Figure 5:**
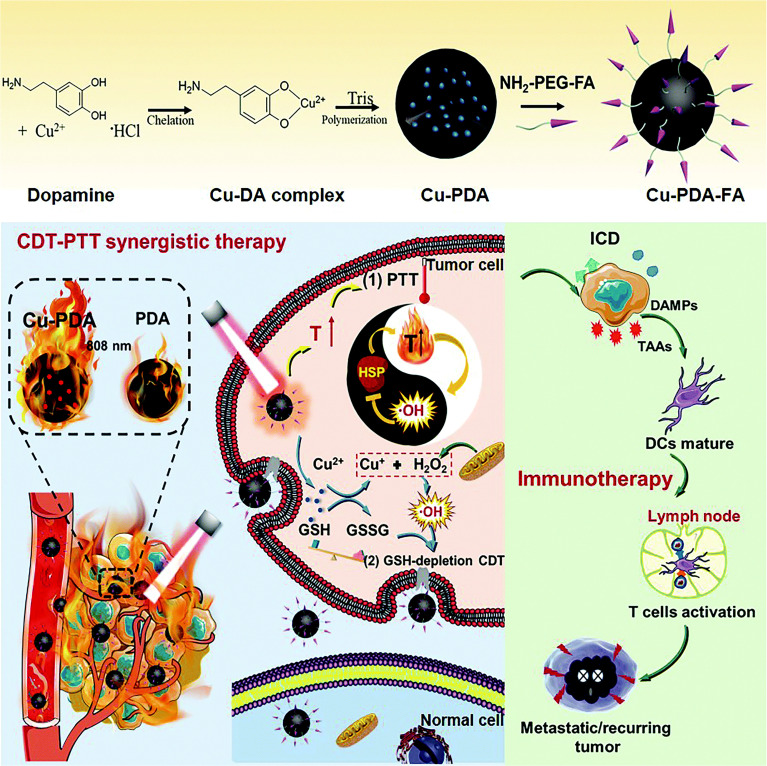
Schematic illustration of Cu–PDA–FA NP synthesis and Cu–PDA–FA NP-mediated PTT/CDT synergistic effect and antitumor immune activation (62). Reproduced with permission from Xu et al., 2022 (62).

PDA has a similar chemical structure to eumelanin and may have similar properties, including NIR-responsiveness, chelation, and drug-binding capability (238). Liu et al. (239) first discovered that PDA has remarkable photothermal conversion ability and applied PDA as PTA to tumors. Xu et al. (62) designed biodegradable folic acid-modified Cu_2+_-chelated PDA nanoparticles (Cu-PDA-FANPs) as an immunogenic cell death (ICD) inducer and multimodal tumor therapy technique (**Figure 5**). Experimental results showed that under the irradiation of 808-nm NIR light, Cu-PDA-FANPs could effectively convert light into heat and cooperate with Cu_2+_-mediated chemical dynamic therapy, promoting a systemic antitumor immune response, which can eliminate tumors *in vivo* and significantly inhibit tumor metastasis.

#### 2.2.3 Other Organic Photothermal Nanomaterials

Melanin is a natural polyphenol substance that can be divided into eumelanin and pheomelanin (240), according to the precursor molecular. Apart from their chelating function and drug-binding ability, natural melanin-based nanomaterials have many beneficial physical and chemical properties, including UV-Vis absorption and excellent PCE. Therefore, the application of natural and artificial melanin-based nanomaterials or melanin-like nanoparticles has achieved remarkable results in the field of biomedicine, especially as antitumor PTT (241, 242). For example, Xie et al. (63) prepared melanin and cellulose nanosheets (OMCNs) using black sesame hell as raw material by facile liquid-phase exfoliation. They then tested the photothermal properties and ability to kill cells of the OMCNs. The extinction coefficient of OMCNs at 808 nm was 3.1 L/g/cm, the PCE was approximately 37.3%, and the OMCNS demonstrated good light stability. The results of cell experiments showed that almost all SMCC-7721 and B16 cells were killed after NIR laser irradiation, indicating that OMCNs have a significant photothermal killing effect on cancer cells and great potential in antitumor therapy *in vivo*. In addition, Kang etal. (64) prepared dual laser-responsive multifunctional melanin-like nanoparticles (MNPs) for PDT, PTT, and chemotherapy, based on the KMnO4-oxidative polymerization of L-3,4-dihydroxyphenylalanine (L-DOPA), pheophorbide a, and Dox, and modified by FA. The results of antitumor experiments showed that after 670-nm and 808-nm laser irradiation, the MNP group showed more extensive damage and apoptosis than the control group, showing great potential for antitumor therapy.

### 2.3 Organic-Inorganic Hybrid Photothermal Nanomaterials

Inorganic photothermal nanomaterials have unique physicochemical properties, such as high molar extinction coefficients, good photothermal conversion rate, excellent photothermal stability, and easy modification; however, their poor biodegradability and potential cytotoxicity limit their use in clinical treatment (91, 243). In contrast, organic photothermal nanomaterials have ideal biodegradability and biocompatibility; however, the photothermal properties of most organic photothermal nanomaterials often require further modification to be used in the treatment of tumors *in vivo* (244, 245). Due to the unsatisfactory effect of inorganic or organic photothermal nanomaterials alone, the application of organic-inorganic composite nanomaterials in PTT has attracted attention. Organic-inorganic composites not only integrate their respective advantages and improve their physical and chemical properties, but also exhibit synergistm (246–248).

Common organic-inorganic composite nanomaterials include core-shell nanoparticles and metal-organic frameworks (MOFs) (249, 250). Odda etal. (65) synthesized surface-engineered iron oxide nanoparticles (α-Fe_2_O_3_NPs) and PEDOT into a novel core-shell photothermal nanoparticle (Fe_2_O_3_@PEDOT-siRNANP), which was loaded with siRNA for synergistic tumor gene therapy and PTT. The experimental results of photothermal conversion performance showed that Fe_2_O_3_@PEDOT-siRNANPs not only had good biocompatibility and water dispersibility but also demonstrated a high PCE (η = 54.3%) in the NIR region. *In vitro* and *in vivo* experiments showed that Fe_2_O_3_@PEDOT-siRNANPs induced greater cancer cell apoptosis and more pronounced tumor suppression after laser irradiation compared with GT or PTT alone. Bai etal. (66) were the first to prepare NIR emission carbon dots (RCDs) based on glutathione (GSH). They then synthesized a novel metal-organic framework nano-platform (RCDS@MIL-100) using RCDs, FeCl_3_, and trimesic acid solutions. In the tumor microenvironment, RCDS@MIL-100 NPs consumed GSH and released Fe^2+^, which could react with hydrogen peroxide to produce hydroxyl radicals. Under the irradiation of 660-nm laser, RCDs showed excellent photothermal conversion ability, promoted a Fenton reaction, and enhanced the therapeutic effect of CDT. The results of antitumor experiments indicated that tumors in the mice of the RCDS@MIL-100 group were completely removed, showing a highly effective antitumor effect.

## 3 Multimodal Therapy for Tumors Based on Photothermal Nanomaterials

Currently, chemotherapy (251), radiotherapy (252), and high-intensity focused ultrasound therapy (253) are widely used and successfully inhibit the growth or spread of tumors and prolong the survival time of patients. PDT (254) has also been shown to have significant advantages in the treatment of non-small cell lung cancer and esophageal cancer. Other treatments, such as PTT (194), immunotherapy (255), gene therapy (256), and magnetothermal therapy (257), have undergone significant research, though most are still in the preliminary clinical stage of research. These emerging tumor treatments have been shown to have ideal anticancer effects in many laboratory and preclinical studies and have broad applications for clinical treatment in the future. For example, ICG, a hydrophobic photosensitizer, is the only NIR imaging reagent approved by the USFDA and has been widely used in the biomedical field, especially for tumor therapy (258, 259). However, ICG has not achieved the eradication of all tumors or the prediction and prevention of metastasis, which is the limitation of single-mode immunotherapy. For example, some cancer cell subsets in heterogeneous tumor tissues may achieve mono-drug resistance to antineoplastic drugs (260). Moreover, long-term use of anticancer drugs often induces multidrug resistance in tumor tissues, which leads to reduced efficacy of chemotherapy (261). Additionally, because of the insensitivity of anoxic cancer cells to ionizing radiation, radiotherapy alone is often unable to achieve an ideal therapeutic effect in a hypoxic tumor environment (262).

Similarly, although PTT has unique advantages, its inherent limitations affect its clinical application. Because the temperature of the tumor site rises to 41–47°C during PTT, necrosis may also occur in the surrounding normal tissue. This leads to the infiltration of pro-inflammatory and immune suppressor cells, triggers a chronic inflammatory response, and promotes immunosuppression through the activation of checkpoint pathways that inhibit T cell responses (263–265). In addition, due to the limited depth of NIR light penetration into the tissue, the tumor cells outside the irradiation area are not completely removed, resulting in possible tumor recurrence and metastasis (263). These limitations inhibit the clinical applications of PTT.

To overcome these barriers of single-mode therapy, combination therapy by integrating two or more treatment modalities has been proposed as a solution (266). Combination therapy is based on synergistic and enhanced interactions between two or more treatments, which tends to produce super additive effects, known as “1+1>2” (267). Therefore, multi-functional nanomaterials are constructed by combining different types of therapeutic agents in a single nanostructure through physical adsorption or chemical binding, which can be used to create multimodal, collaborative therapy for tumors (268–270).

### 3.1 Dual-Modal Therapy Based on PTT

**Figure 6:**
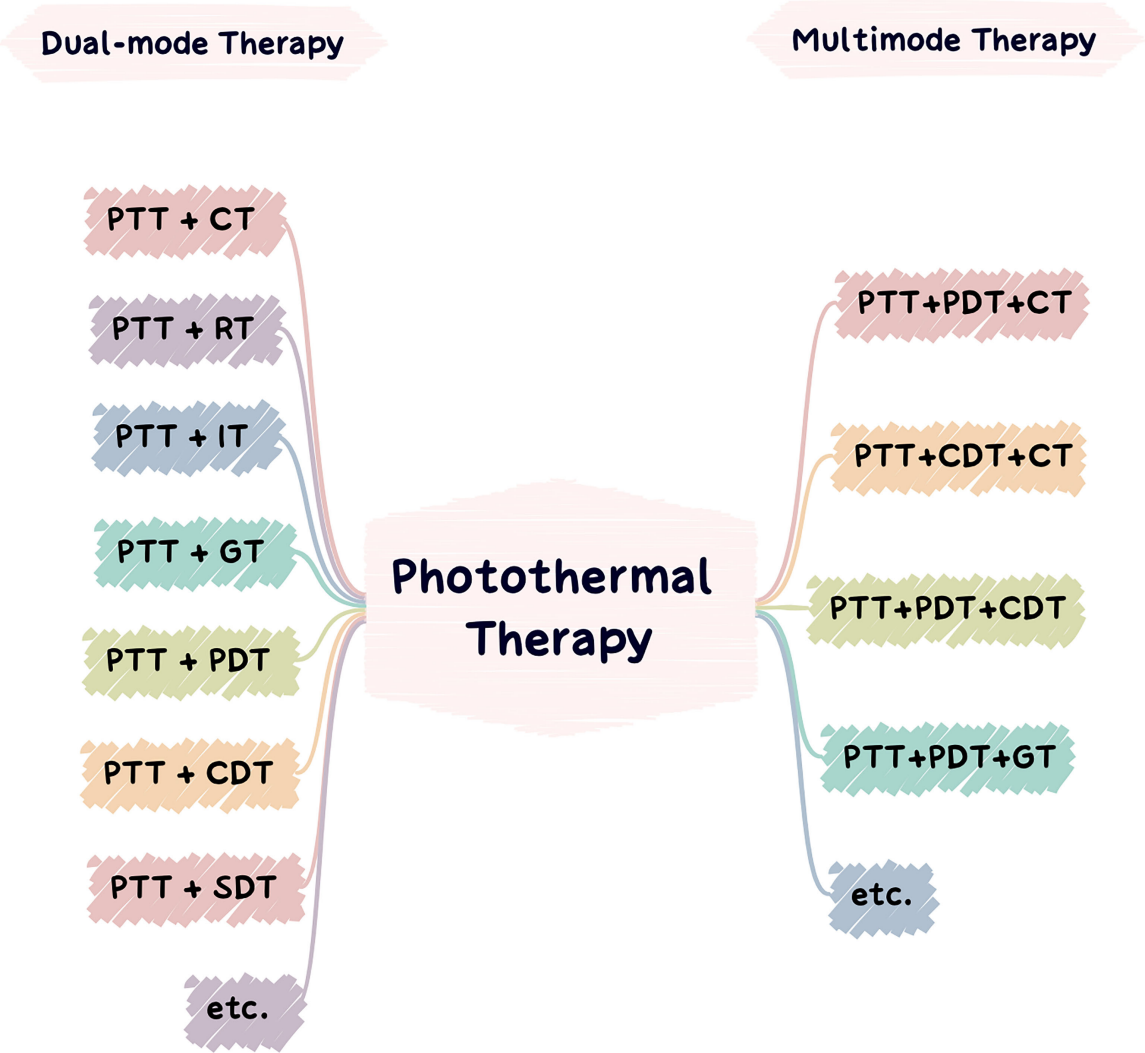
Applications of photothermal nanomaterials in tumor dual-mode therapy (left) and applications of photothermal nanomaterials in tumor multimode therapy (right).

PTT facilitates other tumor treatment methods by its ability to increase the temperature of the tumor site and change the microenvironment. The heat generated during PTT can also promote the intracellular transmission and release of drugs, genes, and immune adjuvants and the disintegration of thermosensitive nanocarriers to enhance the therapeutic effects of chemotherapy, gene therapy, and immunotherapy. It can also accelerate the production of physical/chemical injury factors, such as ROS synthesis, to enhance the efficacy of PDT, sonodynamic therapy (SDT), and CDT based on the principle of oxygen injury. Additionally, due to the increase in temperature from PTT, the vascular permeability of tumor tissue increases, which promotes hemoperfusion, increases oxygen saturation, improves the hypoxic tumor environment, and enhances the efficacy of radiotherapy limited by hypoxia. At the same time, light induces ICD and upregulates tumor immunogenicity, which improves lymphocyte permeability and enhances antitumor immunity (263, 271). In this section, representative studies will be introduced to explain the synergy between PTT and additional therapies (**Figure 6**).

#### 3.1.1 PTT Acts Synergistically by Promoting the Uptake of Therapeutic Agents

Studies have found that the high temperatures produced by PTT can promote the uptake of nanocarriers by tumor cells and accelerate the decomposition of nanocarriers to enhance intracellular drug concentration and cytotoxicity (272). In addition, some anticancer drugs (e.g., cisplatin) exhibit a significantly enhanced ability to kill tumor cells under the high temperature produced by PTT (273). However, due to the thermal expansion of PTT, chemotherapeutic drugs can be more evenly distributed in tumor tissues, enhance heterogeneity, and inhibit tumor drug resistance and metastasis. Wang et al. (274) prepared an intelligent polymer-drug vehicle (MPPD@IR825/DTX) for chemo-photothermal combination therapy, which used poly(ethyleneimine)-poly (ϵ-caprolactone) block polymers as the core and dimethylmaleic anhydride-modified PEG as the shell, encapsulating docetaxel (DTX) and IR825. Compared with the free drug, IR825, MPPD@IR825/DTX nanoparticles exhibited higher temperatures under 808-nm NIR laser irradiation, which increased cytotoxicity and promoted apoptosis of tumor cells more effectively. Furthermore, the results of an *in vivo* antitumor experiment showed that the combination of chemotherapy and PTT has a better effect on tumor eradication, while chemotherapy or PTT alone cannot eliminate the tumor completely.

Similar to combination chemotherapy, PTT can enhance immunotherapy by promoting the uptake of immune adjuvants and the disintegration of nanocarriers by tumor cells. Some PTAs can also be used as immune adjuvants to promote the maturation of dendritic cells and the production of antitumor cytokines (275). After PTT, tumor tissue responds to high-temperature stress and promotes ICD of tumor cells, thereby enhancing antitumor immune responses (276, 277). Wang et al. (278) loaded immune adjuvants, imiquimod (IMQ) and ICG onto amorphous iron oxide nanoparticles (IONs) to design a tumor microenvironment-responsive nanoplatform (IMQ@IONs/ICG). The results of antitumor experiments *in vivo* and *in vitro* showed that IMQ@IONs/ICG had good photothermal conversion ability under 805-nm laser irradiation, induced *in situ* ICD, and cooperated with released IMQ to enhance the antitumor immune response and significantly inhibit tumor metastasis. Compared with the ICG alone group, the primary tumors in the IMQ@IONs/ICG group were completely eradicated after treatment, mesenteric metastasis was significantly reduced, and the survival time of mice was significantly prolonged.

Similarly, PTT can enhance the efficiency of tumor cell uptake by loosening the cell membrane and promoting the release of genes from nanocarriers to enhance gene therapy effects (279). Gene therapy can improve the efficacy of PTT by inhibiting the expression of specific heat shock proteins and overcoming the resistance of cancer cells to thermal damage (280). Xu et al. (281) synthesized a polypyrrole-poly(ethyleneimine)-siILK nanocomplex (PPRILK) gene PTT nanosystem based on the siRNA of integrin-linked kinase (ILK). The results of *in vivo* and *in vitro* experiments showed that after 808-nm laser irradiation, the tumor growth of the PPRILK treatment group was significantly slower and the damage to normal tissue was minimized compared to the laser and gene therapy groups, indicating that the combination of gene therapy and PTT can effectively ablate tumors and inhibit tumor recurrence.

#### 3.1.2 PTT Acts Synergistically by Promoting the Production of Damaging Factors

As an innovative ROS-based cancer treatment, CDT mainly relies on *in situ* Fenton or Fenton-like reactions to generate hydroxyl radical and trigger oxidative damage (282, 283). A kinetic study found that when the temperature increased from 20°C to 50°C, the rate of Fenton reaction was significantly accelerated, giving PTT an unparalleled position in promoting CDT (284). A limitation of PTT is that the expression of HSPs inhibits heat-induced apoptosis (285). HSPs include redress misfolded proteins, such as HSP90 and HSP70, which can alleviate tumor ablation mediated by PTT. Interestingly, studies have found that ROS can effectively inhibit the expression of HSP70, suggesting that CDT, which can generate hydroxyl radicals, is suitable for inhibiting HSP activity and enhancing the efficacy of PTT (286). Huang etal. (287) mixed Ag_2_S nanodot-conjugated Fe-doped bioactive glass nanoparticles (BGN-Fe-Ag_2_S) with PEG double acrylates (PEGDA) and 2,2′-azobis[2-(2-imidazolin-2-yl)propane]-dihydrochloride solution to form a novel light-activated injectable nano-hydrogel (PBFA). The results of *in vitro* experiments showed that under the irradiation of an 808-nm laser, the solution temperature of the PBFA group increased significantly, the concentration of intracellular ROS increased, and the survival rate of tumor cells was much lower than that of the control groups at 33%. Additionally, compared with the control group, the PBFA group could inhibit tumor growth more effectively and showed better biological safety.

The principle of PDT is that photosensitizers are selectively activated and produce cytotoxic ROS through a specific wavelength of light induction, thus inducing tumor cell death (288). It has been reported that PTT produces mildly high-temperatures, can enhance cell membrane permeability to enhance tumor cell uptake of photosensitizer-loaded nanocarriers, and increases intracellular photosensitizer concentration to promote ROS synthesis, enhancing the therapeutic effect of PDT (289, 290). Sun et al. (291) prepared a novel target nanoprobe (Fe/ICG@HA) with porous Fe_3_O_4_ nanoparticles modified by HA and loaded with ICG. The results of antitumor experiments showed that the temperature of the tumor site in the Fe/ICG@HA group increased rapidly to 42.3°C after 808-nm NIR laser irradiation. After 14 days of treatment, the tumor volume of the Fe_3_O_4_ and ICG groups increased slightly, while that of the Fe/ICG@HA group decreased significantly. At the same time, histological examination showed that a large amount of singlet oxygen was produced between tumor cells, indicating that Fe/ICG@HA nanoprobe is a promising nanoplatform for combination PDT/PTT.

In recent years, SDT has been widely revered as a non-invasive tumor treatment method, whose action is to promote acoustic cavitation in tumor cells through the impact of ultrasound on sonosensitizers, thus producing an antitumor effect (292). Additionally, the energy generated by ultrasound can be converted into ROS in the presence of ultrasonic sensitizers (293). Because the lipid arrangement in the biofilm is affected by temperature and membrane permeability increases with temperature, PTT can enhance the SDT cavitation effect (263). Moreover, the ROS and oxygen environment on which SDT depends may enhance PTT/SDT synergism (294). Soratijahromi et al. (295) designed gold/manganese dioxide nanocomposite (Au/MnO_2_ NC) for combination therapy of SDT/PTT. The experimental results showed that under the irradiation of an 808-nm laser and ultrasound, Au/MnO_2_ NC shows excellent photothermal and acoustodynamic conversion ability. Compared with the control group, the production of ROS in the phototherapy/sonotherapy group was significantly increased, which was the most effective in inhibiting melanoma and showed good synergism.

#### 3.1.3 PTT Acts Synergistically by Improving Tumor Hypoxic Environment

PTT can not only promote the generation of ROS to enhance the therapeutic effect of PDT but the mildly high-temperature can also accelerate blood flow to increase the saturated oxygen concentration of blood vessels, which improves the tumor hypoxia environment to promote oxygen production in oxygen-dependent PDT (296, 297). In addition, difficulty in distinguishing normal cells from tumor cells as well as hypoxia-limited ROS production are common pitfalls of radiotherapy (298). Currently, PTT-induced hyperthermia has been observed to accelerate intratumoral blood flow to improve tumor oxygenation (299), thereby reducing hypoxia-induced radioresistance to enhance radiotherapy efficacy (300). In addition, PTT can effectively inhibit the repair of DNA damage caused by X-ray radiation, increase the radiosensitivity of tumor cells, and improve synergism (301, 302). Ni et al. (303) assembled UCNPs coated with manganese dioxide and copper sulfide to create a multifunctional nanoplatform (UCCM) for combined radiotherapy and PTT. The results showed that the manganese dioxide coating produced a large amount of oxygen by interacting with hydrogen peroxide, which can improve the anoxic microenvironment and enhance the efficacy of radiotherapy. Meanwhile, under NIR laser irradiation, the dispersed copper sulfide nanoparticles absorbed light energy and converted it into thermal energy, which significantly inhibited tumor growth. Compared with the radiotherapy or PTT groups, the tumor-bearing mice in the UCCM group had lower cancer cell activity levels and more significant antitumor effects.

#### 3.1.4 PTT Acts Synergistically With Other Methods

In addition to the synergistic effects of PTT with the methods described above, PTT can also promote synergism in other ways. For example, Yang et al. (304) combined superparamagnetic iron oxide nanoparticles and luminescent lead sulfide/cadmium sulfide quantum dots (Pb-based QDs) to create supernanoparticles (SASNs), which verified the feasibility of magnetothermal and photothermal dual-modal hyperthermia. Dual-modal heating with SASN as the heating agent showed an efficient heating output, which was better than magnetothermal and photothermal heating alone. Lu et al. (305) explored the synergism between gas therapy and PTT by designing sulfur dioxide prodrug-doped nanorattles. The experimental results showed that sulfur dioxide had goodPCE, while sulfur dioxide gas had certain cytotoxicity, which could effectively induce tumor cell apoptosis through pH-precise targeting. Compared with the control group without laser irradiation, the expression of pro-inflammatory proteins (Bax, P53, caspase-3) was significantly upregulated in superficial and deep tumors in the combined treatment group after 808-nm laser irradiation, while that of the anti-inflammatory protein, Bcl2, was significantly downregulated, and the apoptosis rate of tumor cells was higher.

### 3.2 Multimodal Therapy Based on PTT

Since the 1960s, the combination of two or more treatment strategies has shown a strong synergistic effect and reduced side effects. Hence, dual-modal or multimodal treatments have skillfully integrated the advantages of a single treatment into one system (282). In contrast to the limited therapeutic effects and possible side effects of single-modal immunotherapy, multimodal synergistic therapy may have the overall advantages of a variety of single-modal immunotherapies and produce higher anticancer effects at lower doses, avoiding high-dose side effects (306). Although dual-modal therapy shows better therapeutic effects than single-modal therapy, multimodal therapies (three or more based on PTT treatments), can further overcome the shortcomings and improve anticancer effectiveness (282). The potential synergistic effect of different treatments has been largely ignored in previous literature because of the complexity of the synergy among treatments. When multiple treatment modes are superimposed, whether contradictions or adverse effects will occur requires further systematic research and analysis. For example, compared with PTT or PDT alone, although their combination can provide a simpler treatment process and more ideal result, it requires higher laser power and irradiation time to initiate synergism. Whether this results in adverse effects on normal human tissue has not been reported (12). Based on the research experience of several groups (307, 308), a variety of therapeutic agents can be assembled in nanocarriers for combination treatment of multiple therapies with higher efficacy and almost no side effects. Below, we provide examples to introduce the research of several multimodal tumor therapy methods.

#### 3.2.1 PTT Combined With PDT and Chemotherapy

Previous studies have shown that drugs, photosensitizers, and PTAs can be integrated into one nanostructure, thus enabling the combination of chemotherapy, PDT, and PTT (309). Because ROS produced during PDT can promote intracellular drug delivery by avoiding uptake of nuclear endosomes, PDT can effectively enhance chemotherapy. Therefore, the combination of PTT/PDT/chemotherapy may be more effective than their dual-modal combinations. Chen et al. (310) synthesized a new multimodal therapy system based on BP nanotablets using Dox as a model drug, which has pH/light-responsive drug release properties. In other words, drug release is further promoted under 808-nm illumination. The results of antitumor therapy *in vivo* showed that, compared with other control groups, the tumor growth inhibition of mice in the three-mode immunotherapy group was the most significant (inhibition rate as high as 95.5%), and the therapeutic effect was significantly enhanced, indicating that the multi-modal combination of PTT/PDT/chemotherapy is feasible.

#### 3.2.2 PTT Combined With Chemotherapy and CDT

The synergistic effect between CDT and PTT has been widely studied and verified (219, 311, 312), but tumor tissues adapt stronger ROS defensive systems at high ROS levels, resulting in poor therapeutic effects from CDT (219, 311, 312). A study (313) found that when PTT andCDT are combined with chemotherapy, this multimodal method can not only overcome the limitation of PTT penetration depth and avoid drug resistance, but also improve the sensitivity of tumor tissue to ROS, achieving significant synergism. Wang et al. (314) designed a nanoparticle based on redox and light-responsiveness (RLR), which consists of ultrasmall iron oxide nanoparticles embedded in an amorphous hollow carbon framework as the core and stacked manganese dioxide flower-like nanosheet structures as the shell. RLR nanoparticles were synergistically treated by manganese dioxide consumption of GSH, iron ion-induced Fenton reaction, PTT, and chemotherapy (Dox). The *in vivo* and *in vitro* results showed that the RLR nanoparticles successfully achieved 99.4% and 99.0% tumor-killing rates, respectively through the synergistic action of CDT, photochemotherapy, and anticancer drugs on a single platform. These results show the potential of the RLR nanoparticle-based platform in multimodal tumor therapy.

#### 3.2.3 PTT Combined With PDT and CDT

**Figure 7:**
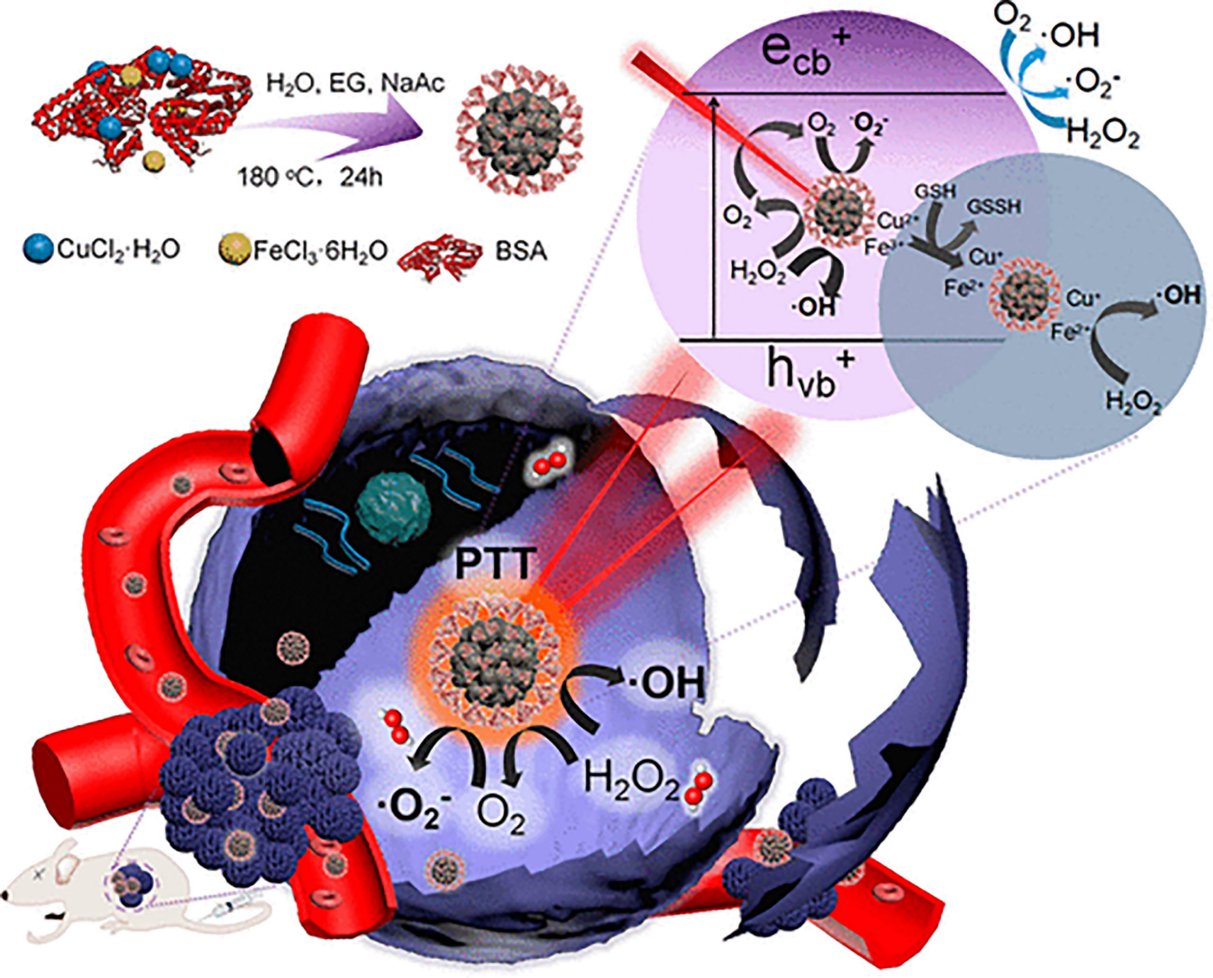
Schematic illustration of synthetic process and therapeutic mechanism of CFNs (317). Reproduced with permission from Liu et al., 2018.

PDT and CDT are mainly ROS-mediated tumor therapy methods. Thus, combining them to construct an antitumor nanoplatform is a promising strategy to improve the antitumor effect (315, 316). Because PTT can improve the hypoxia of PDT, and CDT can inhibit the expression of HSP from PTT, the establishment of a PTT/PDT/CDT multimodal therapeutic platform has attracted attention. For example, Liu et al. (317) prepared biocompatible copper ferrite nanospheres (CFNs) (**Figure 7**). Under 650-nm laser irradiation, the Fenton reaction mediated by copper and iron ions was significant. Meanwhile, CFNs regulated the tumor microenvironment to enhance the therapeutic effect of PDT by promoting the production and consumption of GSH by oxygen. Under the irradiation of 808-nm laser, CFNs exhibited excellent photothermal conversion ability. The experimental results *in vivo* and *in vitro* also showed that when the two wavelengths of laser were irradiated at the same time, almost all tumor cells were killed and the tumors in tumor-bearing mice were eliminated, demonstrating an excellent synergistic antitumor effect.

#### 3.2.4 PTT Combined With PDT and Gene Therapy

Studies have found that some specific types of siRNA (e.g., BAG3-siRNA) used in gene therapy can inhibit the expression of HSPs in cancer cells, which provides the possibility of gene therapy and PTT synergism (318). Among the nanomaterials that have been used to construct multifunctional platforms for tumor therapy and imaging, ultrathin BP nanosheets can not only act as photosensitizers for PDT by effectively promoting the production of large amounts of singlet oxygen (310), but also be widely used in PTT because of their excellent extinction coefficient and PCE (319). Therefore, the construction of a PTT/PDT/gene multimodal therapy nanosystem, based on a BP multifunctional nanoplatform, has aroused interest among researchers (320). Chen et al. (321) used PEG and polyethyleneimine-modified ultrathin BP nanoparticles (PPBP) as a human telomerase reverse transcriptase (hTERT) siRNA delivery system. When irradiated by different wavelengths of laser, PPBP nanoparticles showed excellent PDT and PTT activities, which further promoted the specific release of siRNA for gene silencing antitumor therapy. Experimental *in vivo* and *in vitro* results showed that compared with single-wavelength irradiation, when 660-nm and 808-nm lasers were irradiated together, the expression of hTERT mRNA in mice was significantly reduced and tumor growth was significantly inhibited under the action of PPBP-siRNA nanosheets. After 42 days of treatment, no obvious lung metastases were found. This study demonstrates that the PPBP-siRNA nanoplatform effectively inhibits tumor growth and metastasis through PTT/PDT/gene therapy synergism and verifies the feasibility of this multimodal therapy.

## 4 Conclusion and Outlook

Relying on the rapid development of nanoscience and polymer material technology, the research of photothermal nanomaterials in tumor treatment has made significant progress (322). Although the common inorganic and organic photothermal nanomaterials have great differences in their structures, they all have high PCE and tumor ablation capabilities. Nano phototherapy can not only directly kill tumor cells and reverse drug resistance, but also enhance immune responses (323). In addition, photothermal nanomaterials are becoming increasingly multifunctional through the modification of nanomaterials. Moreover, tumor treatment is becoming increasingly multimodal through the combination of various treatment methods, such as PTT and chemotherapy or PTT and PDT, which have achieved improved therapeutic effects. However, most of these methods are still in the laboratory stage, and these nanomaterials may have defects that limit their clinical application. Thus, the application of photothermal nanomaterials faces many challenges, such as:

1) Due to the different locations of tumor growth and tumor distances from the body surface, the tissue penetrated by laser irradiation has absorbs light, resulting in light weakening or extinction during PTT. In the future, more efforts should be focused on the research and development of PTAs with greater extinction coefficients. In addition, the application method of penetrating the body should also be developed to avoid laser attenuation, such as the administration of PTT at the same time as surgery.

2) The targeting of photothermal nanomaterials in the treatment of tumors, especially in temperature targeting, and avoidance of HSP should be improved. In the future, more attention should be paid to the modification of photothermal nanomaterials to achieve multifunctional uses. For example, treatment based on temperature sensitivity in combination with the application of pH-responsiveness can increase targeting capablities while realizing the multimodality of tumor treatment.

3)Although nanocarriers can co-assemble various therapeutic agents into a single system to build a multifunctional nanoplatform for tumor diagnosis, imaging, and multimodal therapy, the compatibility and composition of therapeutic agents in this technique require further exploration. For example, while PTT and PDT or CDT exert significant synergism, whether the proportion of PTAs and photosensitizer or chemodynamic drugs will inhibit the curative effect of synergistic therapy, as well as the optimal constituent ratio to achieve the best outcome have yet to be determined.

4)PTT is convenient for other treatment modes by virtue of its unique treatment principle; however, the deeper mechanism of the synergy between multimodal treatments remains to be explored. For example, PTT and PDT have an obvious synergistic effect, but the dominant modality has yet to be determined. In addition to the composition ratio of nanodrugs, whether promoting ROS production or alleviating tumor hypoxia is more significant, and how to design drug carriers to best exploit this synergism require further investigation.

5)Finally, the most important aspect is the biosafety of photothermal nanomaterials that remain in the body, which can administer toxicity to the human body. In addition, most solvents and chemicals in these nanomaterials are also harmful to the human body. Although nanomaterials may show low short-term cytotoxicity to cells, tissues, or organs, their long-term cytotoxicity and related immune reactions should be carefully evaluated. One possible method to reduce toxicity is to design biodegradable and cleanable PTAs. However, improving the biodegradability and clearance of PTAs may sacrifice their stability and retention time in the blood, resulting in reduced tumor uptake. Therefore, a balance must be achieved.

In all, photothermal nanomaterials in multimodal tumor therapy present great potential and will become increasingly beneficial with further research.

## Author Contributions

FG contributed to the research retrieval and outline drafting. SC, XS and YT contributed to the research retrieval and drafting and critically revised the manuscript. YL, ZX and SZ critically revised the manuscript. Every author gave final approval and agreed to be accountable for all aspects of the work. All authors have read and agreed to the published version of the manuscript. All authors contributed to the article and approved the submitted version.

## Funding

Financial support from the Projects directly under Jilin Provincial Department of Finance, China (JCSZ2019378-23), and the Scientific Research Project of Jilin Provincial Department of Education, China (JJKH20211215KJ).

## Conflict of Interest

The authors declare that the research was conducted in the absence of any commercial or financial relationships that could be construed as a potential conflict of interest.

## Publisher’s Note

All claims expressed in this article are solely those of the authors and do not necessarily represent those of their affiliated organizations, or those of the publisher, the editors and the reviewers. Any product that may be evaluated in this article, or claim that may be made by its manufacturer, is not guaranteed or endorsed by the publisher.
